# Guided Digital Cognitive Behavioral Program for Anxiety in Primary Care: Propensity-Matched Controlled Trial

**DOI:** 10.2196/11981

**Published:** 2019-04-04

**Authors:** Megan Oser, Meredith L Wallace, Francis Solano, Eva Maria Szigethy

**Affiliations:** 1 Lantern San Francisco, CA United States; 2 Department of Psychiatry University of Pittsburgh Medical Center Pittsburgh, PA United States; 3 Department of General Internal Medicine University of Pittsburgh Medical Center Pittsburgh, PA United States

**Keywords:** anxiety, cognitive behavioral, digital, mobile, primary care, technology

## Abstract

**Background:**

Cognitive behavioral therapy (CBT) is the gold standard treatment for adult anxiety disorders but is often not readily available in a scalable manner in many clinical settings.

**Objective:**

This study examines the feasibility, acceptability, and effectiveness of a coach-facilitated digital cognitive behavioral program for anxious adults in primary care.

**Methods:**

In an open trial, patients who screened positive for anxiety (General Anxiety Disorder-7 [GAD7] score ≥5) were offered the digital cognitive behavioral program (active group, n=593). Primary outcomes included anxiety, quality of life (QoL), and ambulatory medical use over 6 months. Intent-to-treat (ITT) and modified intent-to-treat (mITT) analyses were completed. Subsequently, we compared the outcomes of participants with those of a matched control group receiving primary care as usual (CAU; n=316).

**Results:**

More than half of the patients downloaded the cognitive behavioral mobile app program and about 60% of these were considered engaged, which was defined as completion of ≥3 techniques. The active group demonstrated medium size effects on reducing anxiety symptoms (effect size *d*=0.44; *P*<.001) and improving mental health QoL (*d*=0.49; *P*<.001) and showed significantly improved physical health QoL (*d*=0.39; *P*=.002) and a decreased likelihood of high utilization of outpatient medical care (odds ratio=0.49; *P*<.001). The active group did not significantly outperform the CAU group in anxiety reduction or QoL improvement (*d*=0.20; *P*=.07). However, intent-to-treat analysis showed that the active group had a significantly lower likelihood of high utilization of outpatient medical care than the enhanced CAU group (*P*<.0001; odds ratio=0.09).

**Conclusions:**

A coach-facilitated digital cognitive behavioral program prescribed in primary care is feasible and acceptable. Primary care patients prescribed a digital cognitive behavioral program for anxiety experienced significant improvements in anxiety symptoms, QoL, and reduced medical utilization. This effect was observed even among patients with chronic medical conditions and behavioral health comorbidities. Although the primary outcomes in the active group did not improve significantly more than the CAU group, health care utilization declined, and some secondary outcomes improved in participants who engaged in the program compared to the CAU group.

**Trial Registration:**

ClinicalTrials.gov NCT03186872; https://clinicaltrials.gov/ct2/show/NCT03186872

## Introduction

Untreated anxiety disorders are common and their management is expensive, particularly in medical settings where they can often drive excessive health care utilization [1,2]. When anxiety is identified, treatment is generally administered in primary care; very few patients are referred to specialized mental health care [2] and even fewer receive adequate pharmacotherapy using medications with known efficacy for anxiety disorders or empirically supported cognitive behavioral therapy (CBT) [3,4]. Most cases of clinically significant anxiety in primary care are treated using psychotropic medications, even though CBT is recommended as the first-line treatment [2,4].

Despite the valuable CBT dissemination and implementation work and substantiated models of integrating CB approaches into primary care, scalability remains a problem [5,6]. Digital CB programs are emerging as a solution to this problem [7]. Digital CBT is effective in treating anxiety [8,9] even in non-US primary care settings [10,11]; however, its viability and effectiveness for addressing anxiety in a US primary care setting are not known.

This study is an open trial aimed at determining the feasibility, acceptability, and effectiveness of a digital coach-facilitated CB program for patients receiving routine primary care. In addition, we aimed to compare the primary care sites providing a digital CB program and sites practicing usual primary care. The pragmatic study design allows evaluation of a digital behavioral intervention in routine medical care with minimal disruption of the clinical ecosystem. Although symptom reduction is a key outcome, there is a critical need to address behavioral health factors that contribute to inappropriate medical utilization. Building a scalable approach to effectively manage anxiety symptoms and reducing the negative health and financial impact of unmanaged behavioral health conditions are essential in the era of population health care. We hypothesize that integration of a digital CB program for anxiety within primary care will allow for better access to evidence-based behavioral health care and be effective at reducing anxiety, improving quality of life (QoL), and decreasing high and potentially inappropriate medical utilization. We subsequently compare these outcomes with those of primary care as usual.

## Methods

### Design

All study procedures complied with the ethical standards of the relevant national and institutional committees on human experimentation and with the Helsinki Declaration of 1975, revised in 2008. This prospective pragmatic open trial (Trial Registration: ClinicalTrials.gov NCT03186872) evaluated a digital CB program over a 6-month time period within two primary care sites. The treatment group was then compared to a matched control group from two comparable primary care clinics. Details about the primary care clinics are provided in previous publications [12,13].

### Intervention Condition (Lantern)

The digital CB program Lantern [14] is based on empirically supported CBT protocols for generalized anxiety disorder (GAD) [15,16] with six core skill components (education/awareness, relaxation, thoughts, behavior change/exposure, mindfulness, and habit formation/skill maintenance; Table 1).

**Figure 1:**
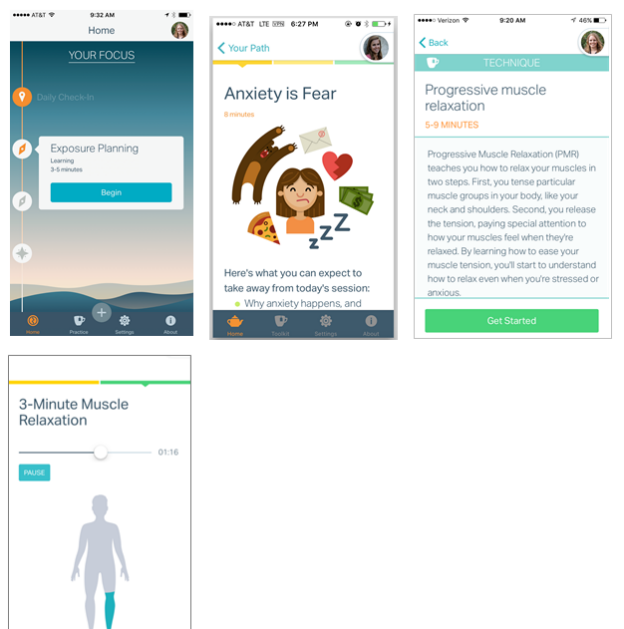
Example of Lantern screenshots.

Lantern was developed in partnership with academic settings, treatment developers, and experts in digital mental health. A scientific advisory board reviewed the entirety of the program to ensure clinical validity. Ideally, users learn all six core skill components to equip themselves with the empirically supported skills to most effectively manage anxiety. The six core skill components of the anxiety program are delivered throughout a series of 40 brief 10- to 15-minute interactive units that introduce a total of 26 techniques, providing behavioral (eg, diaphragmatic breathing) and cognitive (eg, thought-challenging exercises) tools for practice, which users can complete quickly and apply immediately. Patients accessed Lantern via mobile phones (Figure 1).

The Lantern program includes integrated asynchronous texting with a human coach for personalized motivational behavioral coaching. The Lantern coaches provide human support to increase engagement by using motivational techniques, answering questions, monitoring progress throughout the intervention, facilitating goal setting, and reinforcing the content and skills presented to help shape the skill practice into the users’ daily lives. They provide skill coaching through brief text messages through the app. The coaches rely on an internal coaching portal that provides a dashboard for each user. The dashboard shows all user inputs in the program (direct messages to coaches and all content they have completed in the program). Coaches respond to each user a maximum of once per day.

Coaches had backgrounds in health/wellness coaching or education or mental health treatment. All coaches had at least a bachelor’s degree, and the majority of coaches had an advanced degree in their respective fields (master’s level). A doctoral level licensed psychologist supervised the coaches. Coaches were trained in CBT theory and applied techniques, CBT-specific skill-coaching framework, and risk-management protocols. All user messages were reviewed daily for potential risk.

**Table 1:** Core Lantern components.

Core component	Description
Education	Education about anxiety and relationships between thoughts, emotions, and behaviors and what perpetuates anxiety
Relaxation	Relaxation exercises such as diaphragmatic breathing, guided imagery, and progressive muscle relaxation
Cognitive Restructuring	Guidance and techniques to challenge anxiety-maintaining thoughts in the service of developing more adaptive thoughts
Exposure	Further education about the relationship between anxiety and avoidance and how systematic exposure can interrupt the anxiety cycle and facilitate new learning
Mindfulness	Mindfulness explanation and exercises for observing thoughts and emotions without judgement
Habit Formation and Skills Generalization	How to generalize Lantern skills to one’s life and sustain healthy habits

### Comparison Condition

The comparison sites with similar site demographic characteristics to the active practices were offered primary care as usual (CAU), wherein the primary care physician (PCP) managed anxiety as per the standard protocol after positive screening. To enhance CAU at the comparison sites, staff were trained to offer patients a National Institute of Mental Health educational brochure about GAD and its treatment [17] and were provided referral information for mental health clinics with intake availability within 3 months.

### Participants and Recruitment

Patients were enrolled over a 1-year period (2016-2017). Eligibility criteria included age of 20-65 years; ability to speak English; a GAD-7 score ≥5, which is a screening measure part of routine primary care; and access to a mobile phone. At the clinic visit check-in, when a patient scored ≥5 points on the GAD-7, a best-practice alert was generated in the electronic medical record (EMR) to prompt the PCP that the patient is eligible for the digital CB intervention. The PCP decided whether it was appropriate to offer this behavioral health intervention to the patient. If it was appropriate, the PCP referred the patient to the program during the visit. After the patient consented to the study, the PCP ordered the digital CB program via the EMR. Participants could download the mobile app and sign up for the program during the office visit or after the medical appointment. Each participant had access to the digital CB program for 2 years after enrollment.

**Figure 2:**
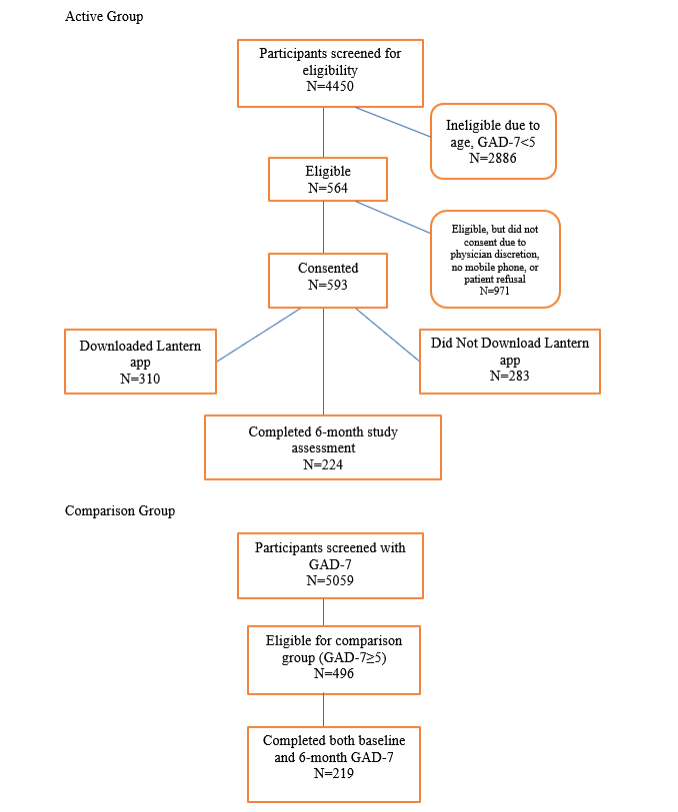
Participant flow sheet.

The comparison group (matched controls) included patients between 20 and 65 years of age who spoke English and had a GAD-7 score ≥5 at screening and a second GAD-7 score 6-months after screening. Figure 2 presents the study recruitment and flow. This study was approved by the University of Pittsburgh Institutional Review Board. The process of patient recruitment and Lantern integration within primary care is detailed in previous publications [12,13].

### Measures

#### Demographics

Data including date of birth, gender, ethnicity, race, and insurance type were abstracted from the EMR.

#### Anxiety Symptom Severity

The GAD-7 scale is a validated self-report questionnaire used to identify probable cases of evaluating the severity of seven diagnostic GAD symptoms occurring in the past 2 weeks [18]. The total scores range from 0 to 21 points. The cutoff of ≥8 points was used to indicate “clinically elevated anxiety.” This cutoff offers the highest sensitivity and specificity balance for GAD and other anxiety disorders and is recommended when evaluating anxiety in primary care with comorbid medical conditions [19,20].

#### Quality of Life

The Short Form Health Survey (SF-12) is a 12-item validated self-report measure assessing health-related QoL (HRQoL) [21]. Results are derived from two component summary scales—Physical Component Summary (PCS-12) and Mental Component Summary (MCS-12)—and scored using the Research and Development (RAND) norm-based methods. Both PCS-12 and MCS-12 summary scores range from 0 to 100 points, with a mean of 50 (SD 10) points, in the general US population. Scores > 50 points represented above-average health status.

#### The Diagnostic and Statistical Manual of Mental Disorders, Fifth Edition, Level 1 Cross-Cutting Symptom Measure

This 23-item self-report measure assesses 13 mental health domains across a range of psychiatric diagnoses [22,23]. We abbreviated the measure to 19 questions assessing depression, anger, mania, anxiety, somatic symptoms, sleep problems, repetitive thoughts and behaviors, dissociation, personality functioning, and substance use. Each item rates how much the person was bothered by a specific symptom during the past 2 weeks (0=none to 4=severe or nearly every day). This measure was only administered to active site patients with baseline GAD-7 scores ≥10 who could be reached by phone within 2 weeks of their screening date.

#### Medical Diagnoses and Utilization

Information on 10th revision of the International Statistical Classification of Diseases and Related Health Problems (ICD-10) code diagnoses during the lifetime, psychotropic medication history in the past 6 months, and medical utilization was obtained from the EMR. A medical complexity score was derived based on the total number of ICD-10 diagnoses in the EMR. Medical utilization includes primary care and specialty medical care ambulatory visits. Information on medical utilization was requested 6 months prior to and 6 months after study enrollment. High utilization was defined as ≥4 outpatient medical visits in the 6 months prior to the study, which was at the 75th percentile.

#### Lantern Helpfulness and Satisfaction Scale

This 14-item self-report scale was administered to patients in the active condition. Respondents rated the helpfulness of and satisfaction with Lantern, length of the program, and the coach and reported the likelihood of recommending Lantern to family/friends.

#### Lantern Utilization

Usage was assessed within the Lantern mobile app for each participant. Usage metrics included the number of units completed, the number of techniques practiced, and the number of days the participant logged into the Lantern mobile app over 6 months. Engagement was defined as completion of ≥3 techniques.

### Statistical Analyses

Data analytic methods were specified *a priori* in the published protocol [13]. Feasibility and acceptability were assessed at the provider and patient levels. Feasibility was assessed using the following early adoption criterion: Among the first 50 patients, at least 50% of eligible participants meeting the inclusion criteria should accept participation in the study. Acceptability was evaluated by patient engagement in the Lantern program and patient satisfaction with the program. As outlined in the published study protocol, to set a rigorous standard for a real-world clinical setting, we set the acceptability threshold as follows: At least 50% of participants that initiate Lantern should complete at least 3 techniques. Descriptive statistics characterize participants’ satisfaction with Lantern.

#### Handling of Missing Data

Approximately 60% of the active group was missing the 6-month data (n=369) because of the manner in which the active group was enrolled and the naturalistic nature of this pragmatic trial. Consequently, we did not perform imputation as per published recommendations [24]. We compared the baseline differences between active group participants who completed the 6-month assessment and those who did not. Compared to active group participants who did not complete the 6-month GAD-7, those who completed the 6-month GAD-7 used more techniques (*P*<.001), were less likely to be high medical utilizers in the prior 6 months (*P*=.05), and were less likely to be female (*P*=.05).

#### Analytic Plan

To test our first aim of a pre-post within-group trial, we characterized baseline characteristics (Table 2) and change scores for continuous outcomes of the full active group sample (N=593). We computed the Cohen *d* value with 95% CIs to summarize within-group effect sizes. For *a priori* hypothesis testing of repeatedly measured outcomes, we used linear mixed-effects models GAD-7, SF12 Mental Health Component (SF12-MHC), and SF12 Physical Health Component (SF12 -PHC) or generalized linear mixed-effects models with a logit link (utilization status). To appropriately model the covariance structure, we included a random subject effect nested within the random clinic effect.

An intent-to-treat (ITT) analysis was performed for the active group participants with baseline and 6-month GAD-7 scores; in addition, a modified ITT (mITT) analysis was performed for participants who completed 3 or more techniques. The mITT analysis facilitates interpretation of the ITT findings by providing better estimates of effects among the patients initiating the intervention [25,26]. Clinically meaningful change and intervention response were calculated for participants with clinically elevated anxiety (GAD-7 cutoff score ≥8). This cutoff score for clinically elevated anxiety offers the highest sensitivity and specificity balance for GAD and other anxiety disorders and is recommended when evaluating anxiety in primary care with comorbid medical conditions [19,20]. Finally, to evaluate the association between Lantern usage, anxiety, and QoL changes, linear mixed model regressions were performed.

#### Propensity Matching

Using the full (unmatched) sample, we performed descriptive statistics, *t*-tests, and chi-square tests to compare the baseline characteristics between active group participants who completed the 6-month assessment and the comparison group. Table 2 describes the difference between the unmatched active and control groups at baseline. Next, propensity score matching was used to develop a matched sample of active and comparison participants with complete baseline data on relevant matching covariates and 6-month follow-up GAD-7 data. When selecting variables for matching, we used an iterative process aimed at striking a balance between attaining a matched sample and retaining power to test effects [27]. In each step of this process, we included variables hypothesized *a priori* to be related to either treatment or outcome (age, gender, race, complexity score, opioid use in past 6 months, and baseline measures of each outcome) and additional variables that differed by group and were endorsed by at least 10% in one group in the resulting matched sample. If the inclusion of one or more (non- *a priori*) variables resulted in >10% loss of sample size, it was not included in order to retain adequate power. For each iteration, the matching procedure effectiveness was assessed by examining standardized mean differences between groups for each matching variable, ensuring all standardized mean differences were less than a “small” effect (Cohen *d*<0.2), and testing for differences in variables that were not used for matching. Final matched variables were age, gender, race, complexity score, medical utilization, baseline GAD-7 scores, baseline SF12-PHC score, baseline SF12-MHC scores, and opioid and antidepressant prescriptions in the past 6 months. Insurance type and presence of diabetes did not match across groups and were not included in order to retain the sample size; however, in the sensitivity analyses, we adjusted the models for these two variables. The final matched sample included 158 active and 158 comparison participants.

Using the final matched sample (N=316), we performed comparison analyses. We computed the Cohen *d* value with 95% CIs to summarize within- and between-group effect sizes. Using this matched sample, we performed ITT and mITT analyses. For hypothesis testing of repeatedly measured outcomes, we used linear mixed-effects models (GAD-7, SF12-MHC, and SF12-PHC) or generalized linear mixed-effects models with a logit link (utilization status). These models included time (baseline and 6 months), treatment (active and comparison), and the time × treatment interaction. To appropriately model the covariance structure, we included a random subject effect nested within the random clinic effect. Clinically meaningful change and intervention response were calculated for participants with clinically elevated anxiety (GAD-7 cutoff score ≥8).

**Table 2:** Matched group comparisons at baseline.

Characteristics		Original unmatched samples						Matched samples					
		Comparison group (n=219)		Active group (n=593)		*P* value		Comparison group (n=158)		Active group (n=158)		*P* value	
Gender - female, n (%)		143 (65.3)		426 (71.8)		.09		100 (63.3)		100 (63.3)		1.00	
Age in years, mean (SD)		47.76 (11.94)		41.49 (13.06)		<.001		45.86 (12.64)		44.03 (13.38)		.21	
Race - white, n (%)		212 (96.8)		537 (90.6)		.01		151 (95.6)		149 (94.3)		.80	
GAD-7^a^, mean (SD)		10.40 (4.9)		11.24 (4.90)		.03		10.46 (5.10)		10.91 (4.97)		.43	
**SF-12^b^** **, mean (SD)^c^**													
	Mental health		35.51 (10.18)		35.55 (9.90)		.97		36.04 (10.27)		35.23 (9.44)		.46
	Physical health		37.04 (12.36)		42.61 (11.59)		<.001		40.21 (11.93)		40.88 (12.27)		.63
Medical complexity score		3.19 (2.44)		2.26 (2.07)		<.001		2.75 (2.28)		2.73 (2.28)		.94	
High utilizer prior to study^d^, n (%)		82 (37.44)		166 (27.99)		.01		43 (27.22)		43 (27.22)		1.00	
Any inpatient encounter in the past 6 months		30 (13.70)		60 (10.12)		.19		14 (8.86)		20 (12.66)		.36	
Any emergency room visit in the past 6 months		53 (24.20)		116 (19.56)		.18		34 (21.52)		31 (19.62)		.78	
**Insurance type, n (%)**													
	Medicare		44 (20.09)		48 (8.36)		<.001		23 (14.56)		15 (9.49)		.23
	Medicaid^e^		64 (29.22)		104 (18.12)		.001		45 (28.48)		29 (18.35)		.05
	Commercial^e^		111 (50.68)		422 (73.52)		<.001		90 (56.96)		114 (72.15)		.01
**Medications in past 6 months, n (%)**													
	Other antidepressants		102 (46.6)		309 (52.11)		.19		80 (50.6)		85 (53.8)		.65
	Tricyclic antidepressants		15 (6.9)		31 (5.23)		.47		8 (5.1)		10 (6.3)		.81
	Benzodiazapines		57 (26.0)		122 (20.6)		.12		42 (26.6)		42 (26.6)		1.00
	Sleep medications		20 (9.1)		49 (8.3)		.80		10 (6.3)		15 (9.5)		.40
	Stimulants		5 (2.3)		6 (1.0)		.29		4 (2.5)		1 (0.6)		.37
	Mood stabilizers		46 (21.0)		56 (9.4)		<.001		26 (16.5)		22 (14.0)		.64
	Buspirone		7 (3.2)		11 (1.9)		.38		4 (2.5)		4 (2.5)		1.00
	Atypical antipsychotics		11 (5.0)		26 (4.4)		.84		4 (2.5)		7 (4.4)		.54
	Opioids		82 (37.4)		100 (16.9)		<.001		31 (19.6)		33 (20.9)		.89
**ICD-10^f^** **codes, n (%)**													
	Depression		91 (41.6)		230 (38.8)		.526		55 (34.8)		70 (44.3)		.11
	Anxiety		92 (42.0)		248 (41.8)		1		61 (38.6)		73 (46.2)		.21
	Substance abuse		7 (3.2)		22 (3.7)		.89		4 (2.5)		6 (3.8)		.75
	ADHD^g^		4 (1.8)		6 (1.0)		.56		4 (2.5)		2 (1.3)		.68
**Medical conditions^h^** **, n (%)**													
	Hypertension		83 (37.9)		118 (19.9)		<.001		54 (34.2)		44 (27.9)		.27
	Chronic GI^i^ condition		66 (30.1)		131 (22.1)		.02		43 (27.2)		45 (28.5)		.90
	Hyperlipidemia		60 (27.4)		93 (15.7)		<.001		39 (24.7)		38 (24.1)		1.00
	Obesity		48 (21.9)		91 (15.4)		.04		28 (17.7)		28 (17.7)		1.00
	Asthma		45 (20.6)		71 (12.0)		.003		27 (17.1)		21 (13.3)		.43
	Diabetes^b^		44 (20.1)		41 (6.9)		<.001		28 (17.7)		14 (8.9)		.03
	Chronic pain		35 (16.0)		83 (14.00)		.55		19 (12.0)		23 (14.6)		.62
	Arthritis		37 (16.9)		54 (9.1)		.003		22 (13.9)		15 (9.5)		.29

^a^GAD-7: Generalized Anxiety Disorder-7.

^b^SF-12: Short Form Health Survey.

^c^For the original unmatched samples, n=216 in the comparison group and n=559 in the active group.

^d^Defined as four or more outpatient medical encounters in 6 months prior to study enrollment.

^e^*P*<.05; cells show mean (SD) for continuous variables or n (%) for categorical variables.

^f^ICD-10: International Statistical Classification of Diseases and Related Health Problems, 10th revision.

^g^ADHD: attention-deficit hyperactivity disorder.

^h^Reporting only those medical conditions with at least 10% prevalence.

^i^GI: gastrointestinal.

## Results

### Participant Characteristics

Baseline characteristics are presented in Table 2. On average, our sample was above the GAD-7 clinical threshold and reported below-average HRQoL (SF-12 < 50 points). About 40% of the sample was diagnosed with depression. The three most commonly prescribed medications in the past 6 months were antidepressants (60.1%), benzodiazepines (26.6%), and opiates (19.6%). Hypertension, chronic gastrointestinal conditions, and hyperlipidemia were the three most prevalent medical conditions. From the Diagnostic and Statistical Manual of Mental Disorders, Fifth Edition, Cross-Cutting Symptom Measure (n=88), the following proportions of patients endorsed domain scores, indicating the need for further assessment into current psychiatric symptoms: anxiety, 84%; depression, 67%; sleep problems, 62%; anger, 58%; personality functioning, 39%; repetitive thoughts and behaviors, 23%; somatic symptoms, 19%; dissociation, 16%; mania, 8%; and substance use, 8%.

### Primary Outcomes

One of two feasibility/acceptability *a priori* criteria were met. The first benchmark was not attained. Among the first 50 patients, less than 50% of the patients who met the inclusion criteria participated in the study. Among the initial cohort of 50 eligible patients, 22% were prescribed Lantern; they had consented to participate in the study and the PCP decided that Lantern would be appropriate for them. This low conversion occurred largely because electronic behavioral health screening was newly launched in primary care, resulting in some technical issues. Among the next set of 50 eligible patients, 36% were prescribed Lantern and among the third set of 50 eligible patients, 44% were prescribed Lantern. There was an incremental increase in study enrollment among the first 150 patients over a 4-week period. The second feasibility/acceptability benchmark was met. More than 50% of participants who initiated Lantern completed ≥3 techniques. There were no adverse events.

### Lantern Usage

A total of 310 participants (of 593, 52.3%) downloaded the Lantern mobile app and completed an average of 6.2 units (SD 9.4, median=2.0, range=0-40 units). Completion of the first 6 units indicated that the participants were exposed to psychoeducation, tracking anxiety cues, motivational interviewing, readiness to change, self-assessment of barriers to change and personal strengths, grounding exercises, applied muscle relaxation, diaphragmatic breathing, mindful walking, and psychoeducation about worry. On average, participants completed 10.4 techniques (SD 17.4, median=4.0, range=0-137) and used Lantern for 16.6 days (SD 21.3, median=9.0, range=0-116) over 6 months. Of the 310 patients who downloaded the app, 27% (n=84) never started the program (ie, completed 0 techniques). Almost 60% (n=183) of participants who downloaded Lantern were considered to be “engaged” in the program (defined as completing ≥3 techniques). Engaged Lantern participants completed an average of 10.3 (SD 10.43) units, which covers automatic thoughts and cognitive reframing. Engaged Lantern participants used Lantern on an average of 26.0 (SD 23.17) days over 6 months.

### Satisfaction and Helpfulness of Lantern

Participants who downloaded Lantern were administered a questionnaire assessing the helpfulness of and satisfaction with Lantern. The satisfaction and helpfulness survey was completed by 122 participants (Multimedia Appendix 1). On a scale of 1-7, with 7 being the highest score, 52% rated the general helpfulness of Lantern as ≥5 and 49% reported their satisfaction with Lantern as ≥5. The majority (65%) reported that the length of the units and overall program was “just right,” 27% felt that they were too long, and 8% reported that the program length was too short. The majority (63.7%) reported that the skillfulness and degree of interest/concern of their Lantern coach was “high.” The majority of study participants (68%) were likely to recommend Lantern to family/friends.

### Anxiety Symptom Severity, Quality of Life, and Outpatient Medical Utilization

The ITT sample included active group participants who completed the 6-month GAD-7 assessment. In the ITT sample (N=224), anxiety symptoms (beta=–2.61; standard error [SE]=0.34; *P*<.001; *d*=–0.44) significantly decreased over time. Mental health QoL (beta=5.71; SE=0.71; *P*<.001; *d*=0.49) and physical health QoL (beta=2.37; SE=0.61; *P*<.001; *d*=0.25) significantly improved over time. From the baseline to 6 months, the odds of being a high outpatient medical utilizer decreased significantly (OR=0.49; beta=–0.72; SE=0.17; *P*<.001) and was 2.04 times (1/0.49) higher than the odds of not being a high utilizer over 6 months.

Using the same mixed-effects model, an mITT analysis was performed using engaged Lantern participants. The engaged Lantern subgroup demonstrated significantly reduced anxiety symptoms with a medium effect size (beta=–3.74; SE=0.49; *P*<.001; *d*=–0.66). In addition, mental health QoL (beta=7.00; SE=1.16; *P*<.001; *d*=0.59) and physical health QoL (beta=3.04; SE=0.97; *P*=.002; *d*=0.39) significantly improved over time. From the baseline to 6 months, the odds of being a high utilizer decreased significantly for engaged Lantern participants (odds ratio=0.15, beta=–1.88, SE=0.61; *P*=.002). The use of Lantern was associated with a 6.67 times increase (1/0.15) in the odds of not being a high utilizer from baseline to 6 months.

### Clinically Meaningful Change and Response Rates

We examined clinically meaningful improvement and deterioration among active group participants who met the clinical threshold for anxiety (GAD-7 score ≥8; n=150). Such an improvement or deterioration was defined as at least a 4-point reduction or increase, respectively, on the GAD-7 score. Among the ITT sample, clinically meaningful improvement was observed in 54% (81/150) of participants. Clinically meaningful deterioration occurred in 8.7% (13/150) of participants. Intervention response (attaining at least 50% reduction in anxiety) was observed in 30% (n=45) of participants. Among the engaged Lantern patients (mITT), 60.8% (45/74) experienced clinically meaningful improvement and 5.4% (4/74) experienced deterioration.

### Relations Between Lantern Usage and Outcomes

Typically, the usage of digital behavioral health interventions is not linearly related to clinical outcomes [28,29]. Examinations of visual plots of usage in relation to anxiety and QoL confirmed that the relationships are nonlinear in this sample, primarily among participants with higher anxiety at baseline. The following post hoc analyses were considered exploratory. Based on quantiles and sample size, we created three categories—0, 1-9, and ≥10—for the number of techniques completed. We explored whether 1-9 or ≥10 techniques were associated with better outcomes (relative to 0 techniques). In the full unmatched sample, the use of 1-9 techniques was significantly associated with improved anxiety (beta=2.30; SE=0.92; *P*=.01) and improved physical health QoL (beta=3.74; SE=1.46; *P*=.01) as compared to the use of 0 techniques. However, the use of ≥10 techniques was not associated with improved anxiety (beta=1.24; SE=0.94; *P*=.19) or physical health QoL (beta=0.96; SE= 1.75; *P*=.58) as compared to the use of 0 techniques. Although not statistically significant, the same pattern emerged, whereby the use of 1-9 techniques was more strongly related to improvements in mental health QoL (beta=3.11; SE=1.84; *P*=.09). Both the visual plots and exploratory analyses suggested that completion of 5-10 techniques was most strongly related to better outcomes. This initial interpretation of the data leads the way for a more nuanced analysis of optimal Lantern usage in the future. All comparative analyses that follow rely on the matched sample.

### Matched Comparisons of Anxiety Symptom Severity, Quality of Life, and Outpatient Medical Utilization

Among the matched active group ITT sample, reduction in anxiety symptoms did not significantly differ from that in the matched comparison group (CAU; Table 3). The matched active group resulted in significantly improved mental health QoL with a medium effect size (*d*=0.50) as well as physical health QoL with a small effect (*d*=0.31). There were meaningful but small effect sizes between the active and control group mental health and physical health composite scores (*d*=0.20 and *d*=0.21, respectively; *P*=.07) between groups. There was a significant time × condition effect on high utilizer status. From the baseline to 6 months, the odds of being a high utilizer decreased significantly more for the active group as compared to the comparison group (OR=0.09, *P*<.001). To facilitate this interpretation, we further examined only the participants who were high utilizers at baseline. Among the 43 high utilizers at baseline in the comparison group, 81% (n=35) remained high utilizers at 6 months. However, among the 43 high utilizers at baseline in the active group, only 35% (n=15) remained high utilizers at 6 months.

Using the same mixed-effects model, an mITT analysis was performed using engaged Lantern participants (n=69; Table 4). The engaged Lantern subgroup demonstrated significantly reduced anxiety symptoms with a medium effect size. Relative to the CAU group (matched comparison group; n=158), there was a larger difference in anxiety reduction, favoring the active group but not reaching significance (*d*=0.22; *P*=.14). The effect size increased in the active group, indicating more improvement in mental health QoL (*d*=0.28; *P*=.06). There was a significant time × condition effect on physical health QoL, and engaged Lantern participants demonstrated significantly more improvement than the comparison group (*d*=0.40; *P*=.01). There was a significant time × condition effect on high utilizer status. From the baseline to 6 months, the odds of being a high utilizer decreased significantly more for the engaged Lantern participants than the comparison group (odds ratio=0.09, *P*<.001). Among the 23 high utilizers at baseline who engaged in Lantern, only 8 (35%) were still high utilizers at 6 months.

### Clinically Meaningful Change and Response Rates Between Matched Groups

Among the patients with baseline GAD-7 scores ≥8, clinically meaningful improvement was observed in 53.5% of the active group (54/101) and 45.8% of the comparison group (44/96). Clinically meaningful deterioration occurred in 11.9% of the active group and 16.7% of the comparison group. Among engaged Lantern patients (mITT), 56.3% (27/48) experienced clinically meaningful improvement. Substantially lower rates of deterioration occurred among engaged Lantern participants (6.3%, 3/48) as compared to 16.7% (16/96) of comparison participants. Intervention response was observed in 27.7% of the active group and 26% of the matched comparison group.

**Table 3:** Anxiety, Quality of Life, and Medical Utilization. Significant findings after adjusting for diabetes and insurance type. Effect sizes of 0.2, 0.5, and 0.8 indicate small, moderate, and large clinically meaningful effects, respectively.

Measures			Mean change (SD)		Effect size (Cohen *d*)				Mixed-effects model				
					Within group (95% CI)		Between group (95% CI)		Beta		SE^a^		*P* value
**Generalized Anxiety Disorder-7 (n=316)**													
	Active	–1.97 (6.06)		–0.32 (–0.55 to –0.10)		0.001 (–0.22 to 0.22)		–0.01		0.66		0.99	
	Control	–1.96 (5.75)		–0.34 (–0.56 to –0.12)									
**Short Form Health Survey Mental Composite Score (n=290)**													
	Active	5.57 (11.08)		0.50 (0.26 to 0.74)		0.198 (–0.03 to 0.43)		2.27		1.27		0.07	
	Control	3.4 (10.77)		0.32 (0.09 to 0.54)									
**Short Form Health Survey Physical Composite Score** **(n=292)**													
	Active	2.81 (9.07)		0.31 (0.07 to 0.55)		0.205 (–0.03 to 0.44)		1.92		1.06		0.07	
	Control	0.95 (9.12)		0.10 (–0.12 to 0.33)									
High utilizer at 6 months (yes/no)		—^b^		—		Odds ratio=0.09		–2.40		0.49		<.001	

^a^SE: standard error.

^b^Not applicable.

**Table 4:** Anxiety, Quality of Life, and Medical Utilization among engaged Lantern participants. Significant findings after adjusting for diabetes and insurance type.

Measures		Mean change (SD)	Effect size (Cohen *d*)		Mixed-effects model		
			Within group (95% CI)	Between group (95% CI)	Beta	SE^a^	*P* value
**Generalized Anxiety Disorder-7 (n=227)**							
	Active	–3.16 (5.38)	–0.59 (–0.93 to –0.24)	0.22 (–0.07-0.50)	–1.20	0.81	.14
	Control	–1.96 (5.75)	–0.34 (–0.56 to –0.12)				
**Short Form Health Survey Mental Composite Score (n=205)**							
	Active	6.47 (11.22)	0.58 (0.18 to 0.98)	0.28 (–0.04-0.60)	3.23	1.71	.06
	Control	3.4 (10.77)	0.32 (0.09 to 0.54)				
**Short Form Health Survey Physical Composite Score (n=207)**							
	Active	4.72 (9.4)	0.50 (0.10 to 0.90)	0.40 (0.08-0.72)	3.79	1.46	.01
	Control	0.95 (9.12)	0.10 (–0.12 to 0.33)				
High utilizer at 6 months (yes/no)		—^b^	—	Odds ratio=0.04	–3.20	0.69	<.0001

^a^SE: standard error.

^b^Not applicable.

## Discussion

### Principal Findings

This pragmatic open trial demonstrated partial feasibility and adequate acceptability among patients who were prescribed Lantern by their PCPs. Although conversion rates from eligibility to consent improved after resolving clinic electronic screening issues, it is plausible that more patients would have enrolled if Lantern was offered systematically to all patients meeting the eligibility criteria. PCPs used their discretion to prescribe Lantern, which impeded our ability to differentiate provider adoption and willingness to prescribe Lantern from patient willingness to participate.

About 73% of patients who downloaded the app started Lantern and nearly 60% engaged in the Lantern program. The average of ≥10 usage days among those who downloaded the Lantern app is comparable to the 10 distinct mobile app use benchmarks followed in digital CBT intervention literature; very few coach-guided CB mobile apps reach this level [30-33]. This above-average engagement in a medical setting is likely related to delivery of Lantern in brief interactive segments, which is aligned with how patients interact with mobile apps via their mobile phones, and coach integration to promote engagement, skills acquisition, and generalization. However, usage patterns suggest that more use is not universally better, which likely represents differences in anxiety severity, types of underlying anxiety disorders, and psychiatric/medical comorbidities. Our exploratory analyses suggest that for some patients, a moderate level of program completion was linked to best clinical outcomes. Thus, it is possible that patients adequately self-dosed the amount of skill training they needed [34-36].

Although there was a significant reduction in anxiety and QoL improvement in the active group receiving Lantern, this reduction did not significantly outperform the matched comparison group in the ITT analysis. One reason for this lack of difference is that patients in the comparison group received CAU, wherein behavioral health resources were provided to PCPs in addition to psychotropic medications prescribed by PCPs. The reduction in anxiety symptoms, the magnitude of effect sizes (│*d* │=0.34-0.35), and proportion of participants attaining clinically meaningful improvement (46%) in primary CAU is about twice that generally observed from care as usual, as reported in the literature [4,37]. This may be because the usual primary care practices were provided with feasible up-to-date behavioral health referral resources, thus potentially enhancing the likelihood that these comparison patients also received behavioral health treatment. Furthermore, the comorbidity of depression and anxiety in this sample may have contributed to the lack of group differences in anxiety and QoL. In addition, the lack of significant differences between the groups is consistent with the results of the 2017 meta-analysis of mobile phone apps targeting anxiety, which showed that stand-alone mobile phone apps did not outperform control conditions in 3 efficacy studies [9]. However, effect sizes of the engaged Lantern participants are comparable to those from a recent investigation of internet-delivered CBT for anxiety in primary care [10]. Furthermore, Lantern may have a greater impact on mitigating clinical worsening of anxiety. Clinical deterioration occurred less often when participants were engaged in Lantern (6.3%) as compared to when they received primary CAU (16.7%).

Interestingly, Lantern had a greater impact on HRQoL than anxiety. Although anxiety has a significant impact on HRQoL, they are sufficiently distinct [38,39]. The mental health component of the SF-12 (HRQoL measure) is a broader outcome capturing many specific behavioral health conditions (depression, anxiety, and loss of behavioral/emotional control) [40,41]. Moreover, the presence of posttraumatic stress disorder (PTSD) or comorbid major depressive disorder has uniquely predicted worse HRQoL among patients with anxiety disorders [37,42]. In a meta-analysis, findings from internet-delivered CBT for anxiety revealed that the largest effects in QoL improvement were found in PTSD, followed by obsessive compulsive disorder, panic disorder, and social anxiety disorder. The smallest effect sizes were observed in GAD [43].

HRQoL improved with patients who used the Lantern anxiety program, which could have occurred via reduction of PTSD symptoms, comorbid depressive symptoms, or other symptoms of anxiety conditions rather than GAD symptoms measured by the GAD-7. In this sample, 44% of patients had a diagnosis of depression in their medical record, and among the subgroup with scores above the anxiety clinical threshold, 67% reported current depression symptoms, 62% reported current sleep difficulties, and 58% reported current anger. Although a specific measure of depressive symptom severity was not used, an improvement in depressive symptoms may have occurred, as prior Lantern evaluations have found significant reductions in depressive symptoms [44,45]. Given the high comorbidity of depression and anxiety, it would be prudent to develop a transdiagnostic program for anxiety and depression conditions in future iterations of Lantern. Furthermore, it is interesting that the sample had high rates of hypertension, chronic gastrointestinal conditions, obesity, and chronic pain—all conditions associated with reduced QoL, anxiety, and depression. Thus, effective management of anxiety or depression could secondarily improve some underlying medical conditions.

The most robust finding is that with Lantern, the likelihood of high outpatient medical utilization was significantly lower than that in a matched comparison group. Both groups showed an average of about three outpatient medical visits within 6 months prior to the study. This is consistent with the average outpatient utilization among patients with anxiety disorders who are untreated or undertreated [1]. A total of 62%-65% of Lantern participants were no longer high medical utilizers in the follow-up period, whereas the comparison group had more than twice the number of patients who remained high utilizers at 6 months. Although Lantern can improve self-reported QoL and anxiety, its most significant impact is in reducing anxiety-related behavior (outpatient medical visits). A subsequent evaluation will focus on high medical utilizers.

### Limitations

This was an open pre-post pragmatic trial. Careful propensity matching allowed for the most rigorous comparison in the absence of randomization. However, it was important to show what results were possible in a pragmatic trial with little research infrastructure to be able to generalize our findings for real-world settings. Information provided to PCPs was minimal. PCPs may benefit from better guidelines for selecting appropriate candidates for Lantern. Due to the absence of a research infrastructure to guide study recruitment and retention, there was a high percentage of missing outcome data in the active group, which limits interpretation of the findings but still offers value as a feasibility trial. We are not aware of how frequently PCPs referred to psychotherapy or prescribed psychotropic medications at the comparison sites. When comparing the minimally engaged subsample (mITT group) to the matched controls, it is important to remember that the comparison group was matched to the full ITT sample and not the mITT sample. Thus, it may be that more likely that patients who showed improvements continued to use the app as a source of survival bias. However, the purpose of the mITT evaluation was to facilitate interpretation of the effect size in relation to the ITT group. There is also a possibility of a “digital placebo” effect, whereby the benefits may be due to placebo engagement in the mobile app intervention rather than the active components of the CB program itself [46]. This is particularly likely in the subgroup of engaged users and can be tested in future investigations using a different study design. The results should also be interpreted in light of the predominately white sample and rely on the ICD-10 codes for identifying comorbidities.

### Conclusions

Lantern demonstrated moderate effectiveness for addressing anxiety symptoms over 6 months, but not more than primary CAU. Lantern led to a robust decrease in the number of high outpatient medical utilizers among primary care patients with other morbidities such as depression, obesity, hypertension, chronic pain, chronic gastrointestinal conditions, and use of psychotropic medications. The surprising effectiveness seen in primary CAU may have suppressed group differences typically observed with standard CAU. However, with the minimal research infrastructure of this pragmatic study and limited resources to optimize PCP uptake and effectiveness of prescribing Lantern, these outcomes are meaningful from a population health and medical cost-offset perspective. This may be the first study to show that a coached CB program delivered via a mobile app provided in primary care reduces outpatient medical utilization compared to a matched control group. Whether this decreased utilization was diverted to other types of medical utilization (eg, emergency department visits) remains unknown. Future analyses of 12-month follow-up data will provide information about the extent to which Lantern has an impact on improving appropriate healthcare utilization.

## Appendix

Multimedia Appendix 1 Lantern satisfaction and helpfulness questionnaire (n=77) results.

## Abbreviations

CAU: care as usual
CBT: cognitive behavioral therapy
EMR: electronic medical record
GAD: Generalized Anxiety Disorder Questionnaire
HRQoL: health-related quality of life
ICD-10: International Statistical Classification of Diseases and Related Health Problems, 10th revision
ITT: intent to treat
MCS: mental composite score
MHC: Mental health composite
mITT: modified intent-to-treat
PCP: primary care physician
PCS: physical composite score
PHC: physical health composite
PTSD: posttraumatic stress disorder
RAND: Research and Development
SE: standard error
SF-12: Short Form Health Survey
QoL: quality of life
