# Supplementary figures and images for: Guided Digital Cognitive Behavioral Program for Anxiety in Primary Care: Propensity-Matched Controlled Trial
Source: JMIR Ment Health. 2019 Apr 4;6(4):e11981. doi: 10.2196/11981 (PMC6470461; doi:10.2196/11981)

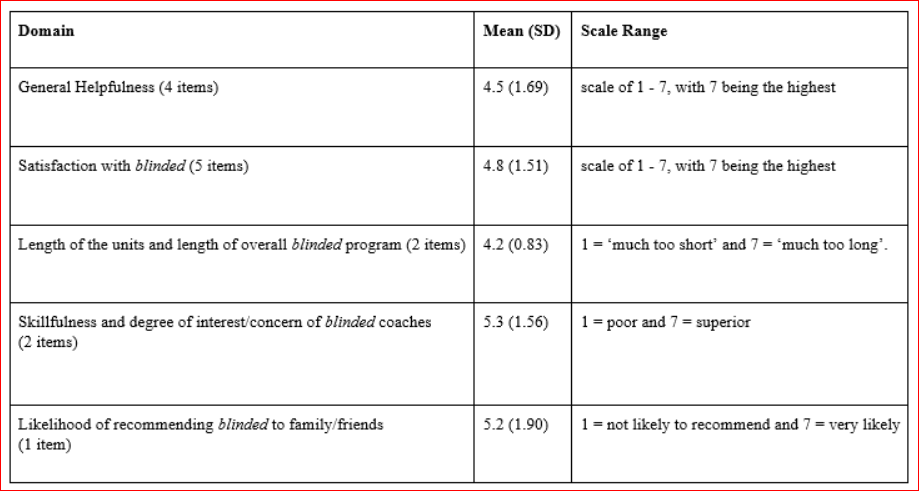

Supplement: Multimedia Appendix 1 [file mental_v6i4e11981_app1.PNG]
